# microRNA-10b Is Overexpressed and Critical for Cell Survival and Proliferation in Medulloblastoma

**DOI:** 10.1371/journal.pone.0137845

**Published:** 2015-09-22

**Authors:** Rekha Pal, Stephanie Greene

**Affiliations:** Department of Neurological Surgery, Children’s Hospital of Pittsburgh, University of Pittsburgh Medical School, Pittsburgh, Pennsylvania, United States of America; University of Saarland Medical School, GERMANY

## Abstract

This study demonstrates the effects of miRNA-10b on medulloblastoma proliferation through transcriptional induction of the anti-apoptotic protein BCL2. Using a cancer specific miRNA-array, high expression of miRNA-10b in medulloblastoma cell lines compared to a normal cerebellar control was shown, and this was confirmed with real time PCR (RT-PCR). Two medulloblastoma cell lines (DAOY and UW228) were transiently transfected with control miRNA, miRNA-10b inhibitor or miRNA-10b mimic and subjected to RT-PCR, MTT, apoptosis, clonogenic assay and western blot analysis. Transfection of miRNA-10b inhibitor induced a significant down-regulation of miRNA-10b expression, inhibited proliferation, and induced apoptosis, while miRNA-10b mimic exerted an opposite effect. Inhibition of miRNA-10b abrogated the colony-forming capability of medulloblastoma cells, and markedly down-regulated the expression of BCL2. Down-regulation of BCL2 by antisense oligonucleotides or siRNA also significantly down-regulated miRNA-10b, suggesting that BCL2 is a major mediator of the effects of miRNA-10b. ABT-737 and ABT-199, potent inhibitors of BCL2, downregulated the expression of miRNA-10b and increased apoptosis. Analysis of miRNA-10b levels in 13 primary medulloblastoma samples revealed that the 2 patients with the highest levels of miRNA-10b had multiple recurrences (4.5) and died within 8 years of diagnosis, compared with the 11 patients with low levels of miRNA-10b who had a mean of 1.2 recurrences and nearly 40% long-term survival. The data presented here indicate that miRNA-10b may act as an oncomir in medulloblastoma tumorigenesis, and reveal a previously unreported mechanism with Bcl-2 as a mediator of the effects of miRNA-10b upon medulloblastoma cell survival.

## Introduction

Medulloblastoma is the most common malignant brain tumor in children, affecting 2 per 100,000 children in the United States and worldwide, and comprising approximately 30% of new pediatric brain tumor diagnoses. Patients with clinical standard-risk disease have 5-year survival rates that approach 80%, while those with high-risk disease have much lower survival rates. The side effects of the treatment course, which involves surgery, craniospinal radiation therapy and intensive chemotherapy, are considerable. Attempts to predict outcome based upon pathologic subtype, genetic mutations, and protein expression have been largely unsuccessful. The development of new therapeutic targets is essential to increasing survival rates and reducing therapy-related side effects.

Medulloblastoma represents a heterogeneous group of tumors that has recently been categorized into 4 subgroups on the basis of clinical factors and molecular signature [1]. The WNT subgroup has a very good prognosis, and may benefit from treatment modification to minimize morbidity. No cell line has been developed that represents this subgroup well. Up to 50% of tumors in the SHH subgroup are pathologically classified as desmoplastic or nodular medulloblastomas. While the DAOY cell line was derived from a desmoplastic medulloblastoma, the cytogenetics are atypical for the SHH subgroup. Group 3 tumors carry a very poor prognosis, and metastases are often present at the time of diagnosis. There is a need to accurately identify these tumors soon after surgical resection, and to develop treatments tailored to biomarkers specific to this tumor subgroup. Cell lines such as D341, probably D283 (which also has an isochromosome 17), UW402, UW228 [2], and D425 are MYC-amplified and most closely resemble group 3 tumors [3]. Group 4 tumors most frequently have an isochromosome 17q, are often metastatic at the time of diagnosis, and have an intermediate prognosis. This is the most common, and the least understood, tumor subgroup [1].

MicroRNAs (miRNAs) are small, highly conserved RNA molecules that regulate gene expression post-transcriptionally, by inhibiting the translation of target mRNAs into protein or by degrading target mRNAs [4]. Aberrant expression of miRNAs has been shown to play an important role in the pathogenesis and metastasis of various malignancies [5–7]. Oncogenic miRNAs have been shown to promote carcinogenesis by targeting tumor suppressors such as cell cycle regulators and pro-apoptotic genes [8,9]. Such oncomirs are potential therapeutic targets.

Recent miRNA expression profiling studies revealed a number of miRNAs implicated in medulloblastoma initiation and progression through effects on genes and proteins responsible for cell proliferation or cell death [10,11]. Overexpression of miRNA-10b associated with tumorigenesis has been reported in a number of tumor types, including low-grade glioma [12–16], breast cancer [14], and esophageal cancer [17]. Increased expression of miRNA-10b has been shown to promote proliferation by inactivating the expression of pro-apoptotic genes [12,13,17,18,19]. Gabriely et al have shown that human glioma growth is controlled by miRNA-10b directly targeting BCL2L11 / Bim, a proapoptotic protein commonly activated in response to chemotherapy [16]. These studies strongly suggest that miRNA-10b is an oncomir, and plays an important role in oncogenesis by controlling multiple cellular pathways.

This study demonstrates the effect of miRNA-10b on medulloblastoma growth and proliferation through transcriptional induction of BCL2, a cellular proto-oncogene and tumor promoter. A loss of function approach was used to elucidate miRNA-10b function in medulloblastoma. In this study, the effects of ABT-737 and ABT-199, potent specific inhibitors of BCL2, on miRNA-10b expression were examined. High levels of miRNA-10b were found to correlate with multiple tumor recurrences and low survival in patient samples. Collectively, our data demonstrate that BCL2 is an important mediator of the effects of miRNA-10b in medulloblastoma, and suggest a potential new therapeutic approach in the treatment of medulloblastoma.

## Material and Methods

### Cell lines and reagents

DAOY, D341 and D283 human MB cell lines were purchased from American Type Culture Collection (Manassas, VA). UW228, UW402 and UW426 were the gifts of Dr John R. Silber (University of Washington, WA).[2] D384 Med and D425 Med cell lines were the gifts of Dr Darell D. Bigner (Duke University, Durham, NC).[3] All cell lines were passaged for no more than 6 months after receipt or resuscitation, and so authentication from the sources was accepted. No de novo cell lines were used or generated for this project. MB cell lines except D341 were cultured in DMEM medium supplement with 1X penicillin/streptomycin and 10% fetal bovine serum (FBS) at 37°C and 5% CO_2_. The D341 cell line was cultured in DMEM with 20% FBS with 1% penicillin/ streptomycin at 37°C and 5% CO_2_. Antibodies against BCL2 were purchased from Cell Signaling Technology, Inc, and against β- actin from Sigma-Aldrich. ABT-199 and ABT-737 were purchased from Active Biochem and dissolved in DMSO for stock solution. DAOY and UW228 cells were treated with DMSO (control group) or with varying concentrations of ABT-737 and ABT-199 in DMEM supplemented with 1X penicillin/streptomycin and 10% fetal bovine serum (FBS) at 37°C and 5% CO_2_ for 2 days. Cells were subjected to RNA extraction, MTT and apoptosis assays.

### Patient samples

Patient medulloblastoma samples were collected from the University of Pittsburgh Brain Tumor Bank after University of Pittsburgh Institutional Review Board approval was obtained. Informed consent was not obtained as the samples were analyzed restrospectively after being stored in the Brain Tumor Bank. Thirteen medulloblastoma specimens were analyzed for this study. All samples were stored in liquid nitrogen immediately after resection and stored at -80°C until use. The ages of the patients included in this study ranged from eight month to 26 years. The majority of patients were male (70%), and 70% of these patients died of their disease (Table 1). Normal cerebellar samples from autopsied patients were obtained from the neuropathology Reference RNA (Ambion) to ensure that there was no difference in miRNA production between normal human cerebellum and the pooled brain tissue from multiple brain regions used for the First Choice Human Brain Reference RNA. No difference was found (data not shown).

**Table 1:** miRNA-10b expression correlates with survival and recurrence in human medulloblastoma patient samples. Human biopsy samples were subjected to RNA extraction followed by generation of cDNA, and subjected to RT-PCR for analysis of miRNA-10b expression. The fold expression of miRNA-10b was compared to the normal cerebellar control. miRNA-10b expression was correlated with the patients’ clinical and pathological history.

Patient #	Gender	Age at surgery	Time to recurrence	#Recurrences	Time to Death	MIB-1	Pathologic subtype	Molecular subgroup	miRNA-10b expression
1	F	3y10m	16m	4	3y11m	35%	Classic	Group 3	1.947
2	M	34m	13m	1	2y1m	45%	Desmoplastic	SHH	0.0816
3	M	25	53m	1	5y4m	80%	Desmoplastic	SHH	1.043
4	M	12	31m	1	4y9m	43.60%	Classic	Group 4	0.414
5	F	16	27m	4	7y5m	57.10%	Classic	Wnt	**198.400**
6	M	7y9m	none	0		60%	Desmoplastic	Group 4	2.472
7	F	2y11m	none	0		65.40%	Desmoplastic	Wnt	**13.056**
8	M	13m	none	0		40–50%	Desmoplastic	SHH	0.233
9	M	4y5m	16m	1	1y8m	33.30%	Classic	Group 3	7.223
10	M	8m	14m	3	3y2m	36.10%	Desmoplastic	SHH	0.260
11	M	16	none	0		34.32%	Classic	Group 4?	**52.740**
13	F	26	6m	5	4y8m	30%	Classic		589.254
14	M	17m	4m	2	8m	40%	Classic		0.448

### RT-PCR analysis for miRNA expression

Total RNA was extracted from the cell lines, normal cerebellar control samples, and patient medulloblastoma samples using the Qiagen miRNAeasy Mini Kit and the manufacturer’s protocol. Total RNA was converted into miRNA first strand cDNA using the RT^2^ miRNA First Strand kit for the DAOY and D341 cell lines, human medulloblastoma samples, and the human cerebellar control samples. Quantitative reverse transcription polymerase chain reaction (RT-PCR) was performed on a Bio-Rad iCycler using 25 ul of cDNA and RT^2^ SYBR Green qPCR master mix per well in a 96-well miRNA Human Cancer array. A three-step cycling program (10 minutes at 95°C, 40 cycles of 15 seconds at 95°C, 30 seconds at 60° and 30 seconds at 72°C) was used. The resulting threshold cycle values for all wells were exported to an Excel spreadsheet for use with the miRNA PCR Array Data Analysis Template. Expression analysis of the 88 cancer-specific miRNA sequences selected for the Human Cancer RT^2^ miRNA PCR array was performed. All materials were purchased from SA Biosciences unless otherwise indicated.

For the determination of miRNA levels of miRNA-10b, miRNA-17 and miRNA-20b, the average threshold cycle (C_T_) for expression of each miRNA was determined from triplicate reactions, and data were analyzed using the difference between threshold PCR cycles values for target and control genes (ΔC_T_). miRNA expression was normalized to internal control RNA such as SNORD 47 and SNORD 48 using the ΔC_T_ values. Each result was calibrated to the cerebellar control to give the ΔΔC_T_ value, in which the control had a ΔΔC_T_ value of 0. The fold target miRNA expression compared with the calibrated value is given by the formula: 2^- ΔΔCT^.

### Transfection studies

DAOY and UW228 cell lines were transfected with control miRNA, miRNA-10b inhibitor (miRNA-10b-AS) (GeneCopoeia, Rockville, MD), or miRNA-10b oligonucleotide (Qiagen) using the Lipofectamine RNAiMAX reagent (Invitrogen) for the control miRNA and anti-miRNA-10b as per the manufacturer’s guidelines. For the miRNA-10b mimic studies, the transfection was performed with the control miRNA or miRNA-10b oligonucleotide using the LTI reagent (Mirus, Madison, WI). All transfections were carried out in 24-well plates using 2 x 10^4^ cells / well in 500μl of medium. Protein and RNA from the transfected cells were extracted for analysis of miRNA-10b, BCL2 and Mcl-1 expression. For the experiments assessing the effect of silencing of BCL2, DAOY cells (2 x 10^4^ cells /well) were plated in 24-well plates 24 hours before transfection. Plated cells were transfected with Dicer substrate 27-mer duplexes targeting BCL2 mRNA and scrambled universal negative control RNA duplex (Origene, Rockville, MD). On the day of transfection, 500 μl of fresh medium and 10nM of siRNA were mixed with the transfection reagent (Lipofectamine RNAiMAX reagent) as per the manufacturer’s instructions and added to each well. The cells were harvested for RNA and protein extraction after 48 hrs for RT-PCR and Western blot analysis.

### Western blot analysis

Briefly, the cells were lysed with 1X radioimmunoprecipitation assay buffer (Santa Cruz Biotechnology Inc) with a cocktail of protease inhibitors, sodium benzoate and phenylmethylsulfonyl fluoride. Cell lysates were subjected to 10% sodium dodecyl sulfate-polyacrylamide gel electrophoresis, and transferred to polyvinyl difluoride membranes (Bio-Rad Laboratories). The blots were incubated with the respective antibodies for protein expression and the immune complexes were detected using enhanced chemiluminescence (Amersham).

### Cell proliferation assays

Cell proliferation was measured by MTT assay (Cayman Chemical Company, MI). Briefly, medulloblastoma cells were plated in six-well plates and transfected with control miRNA, anti-miRNA-10b-, or miRNA-10b mimic. After 48 hours, 5000 transfected cells / well were seeded in 96-well plates in 100 μl of medium. Ten μl of MTT reagent were added to each well, and the cells were incubated for 3 hrs at 37°C. Absorbance was measured at 570 nm using a microplate reader according to the manufacturer’s recommendations. Relative cell number was calculated by normalizing the absorbance to that of untreated cells.

### Apoptosis assay

Apoptosis was analyzed using a cell death detection ELISA kit (Roche Diagnostics, Indianapolis, IN) based on a photometric enzymatic immunoassay for the quantitative *in vitro* determination of cytoplasmic histone-associated DNA fragments as described ^11^. Briefly, equal numbers of medulloblastoma cells were seeded in six-well plates and transfected with the control miRNA, miRNA-10b-AS or miRNA-10b mimic. The cytoplasmic fraction was collected from each sample after 48 hours of transfection as per the manufacturer’s protocol. The cytoplasmic fractions were incubated with biotin- labeled monoclonal anti-histone antibody, followed by incubation with peroxidase-conjugated monoclonal anti-DNA antibody. Color development was assessed with ABTS substrate measured at 405 nm against substrate solution as a blank control.

### Clonogenic assay

To determine the ability of medulloblastoma cells to clonally expand and create colonies, cells were transfected with control miRNA, miRNA-10b-AS or miRNA-10b mimic for 48 hours, and seeded in 6-well plates (3000 cells/ well) in triplicate. After 7 days of incubation at 37°C, the plates were gently washed with PBS. The colonies were stained with Hema3 Manual Staining Systems (Fisher Scientific). The plates were scanned, and images were represented in JPG format and placed into figures using Adobe Photoshop Version 7.0 Professional (Adobe Systems).

### Statistical analyses

Each experiment was repeated in at least triplicate, and all quantitative data are presented as mean plus or minus SD. Statistical differences were determined by Student’s *t* test. The results were considered statistically significant with *p* values < .05.

## Results

### miRNA-10b is highly expressed in medulloblastoma cells

**Fig 1:**
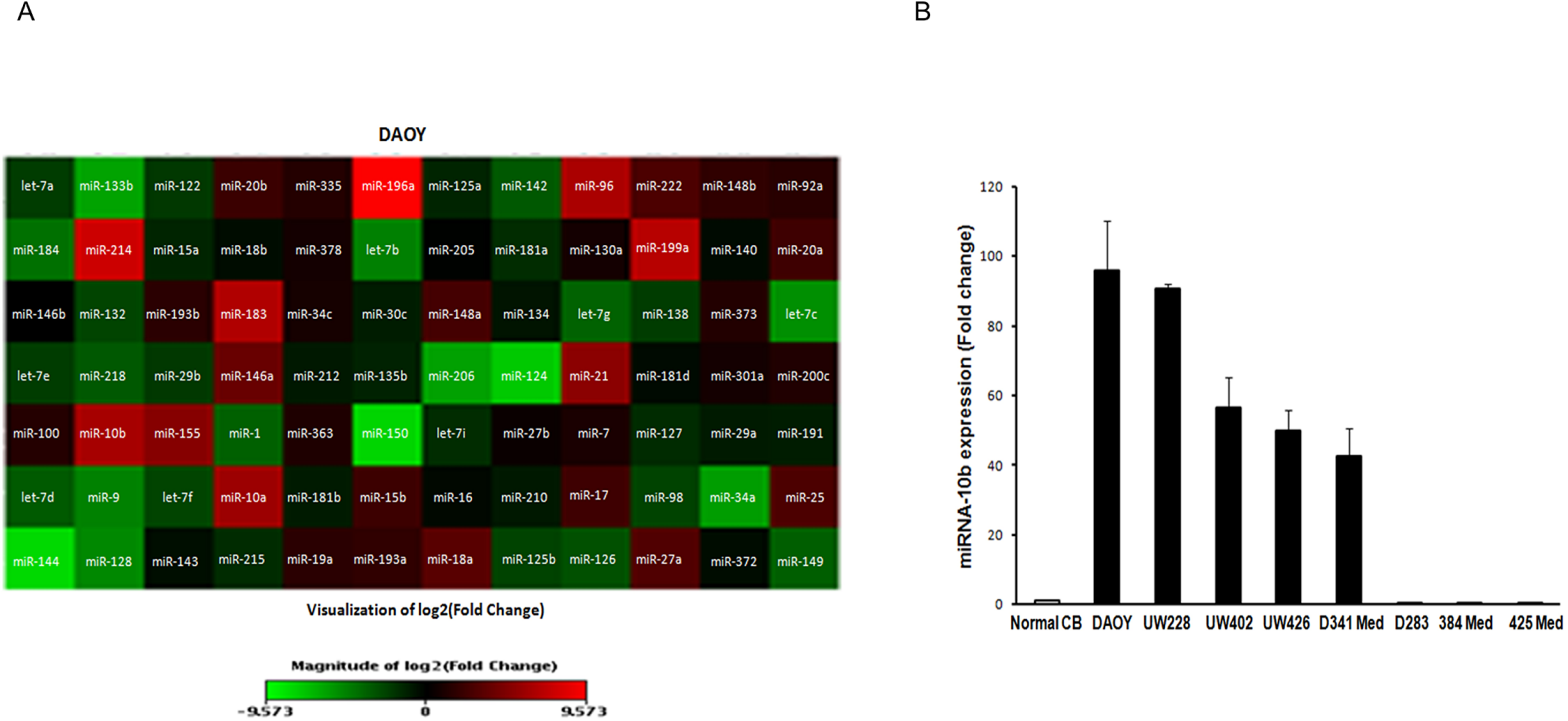
miRNA-10b is highly expressed in medulloblastoma cells. Total RNA was extracted from medulloblastoma cells and subjected to RT-PCR analysis. (A) Oncomir microarray using quantitative RT-PCR in DAOY cells compared to normal cerebellum and FirstChoice® Human Brain Reference RNA. MicroRNA heat map profile. (B) Increased expression of miRNA-10b in the majority of medulloblastoma cell lines (DAOY, UW228, UW402, UW426 and D341 Med).

To investigate the profiling of oncomirs in medulloblastoma pathobiology, a cancer-specific miRNA-array was performed. From the oncomir microarray, miRNA-10b was identified as one of the most highly and significantly upregulated miRNAs in medulloblastoma cell lines when compared to a normal cerebellar control (Fig 1A left panel). We decided to investigate miRNA-10b expression further because miRNA-10b has been reported to be strongly associated with cancer ^13,18,19^, to play a role in cellular proliferation and survival^16^, and because its role in medulloblastoma is still unknown. miRNA-10b is not expressed in normal brain. While HRK was also significantly upregulated, its pro-apoptotic role made it a less attractive subject for further study of proliferation. To confirm the high expression of miRNA-10b, we analyzed its expression by quantitative RT-PCR in eight medulloblastoma cell lines. We found that miRNA-10b expression was significantly upregulated (42–100 fold) in five medulloblastoma cell lines (DAOY, UW228, UW402, UW426, and D341 Med) (Fig 1B) compared to the control. Three medulloblastoma cell lines (D283, 384 Med, and 425 Med) from this study showed no expression of miRNA-10b. The DAOY (SHH subgroup) and UW228 (Group 3) medulloblastoma cell lines showed the highest miRNA-10b expression, and so these cell lines were utilized for further functional studies targeting miRNA-10b.

### miRNA-10b induces medulloblastoma proliferation

**Fig 2:**
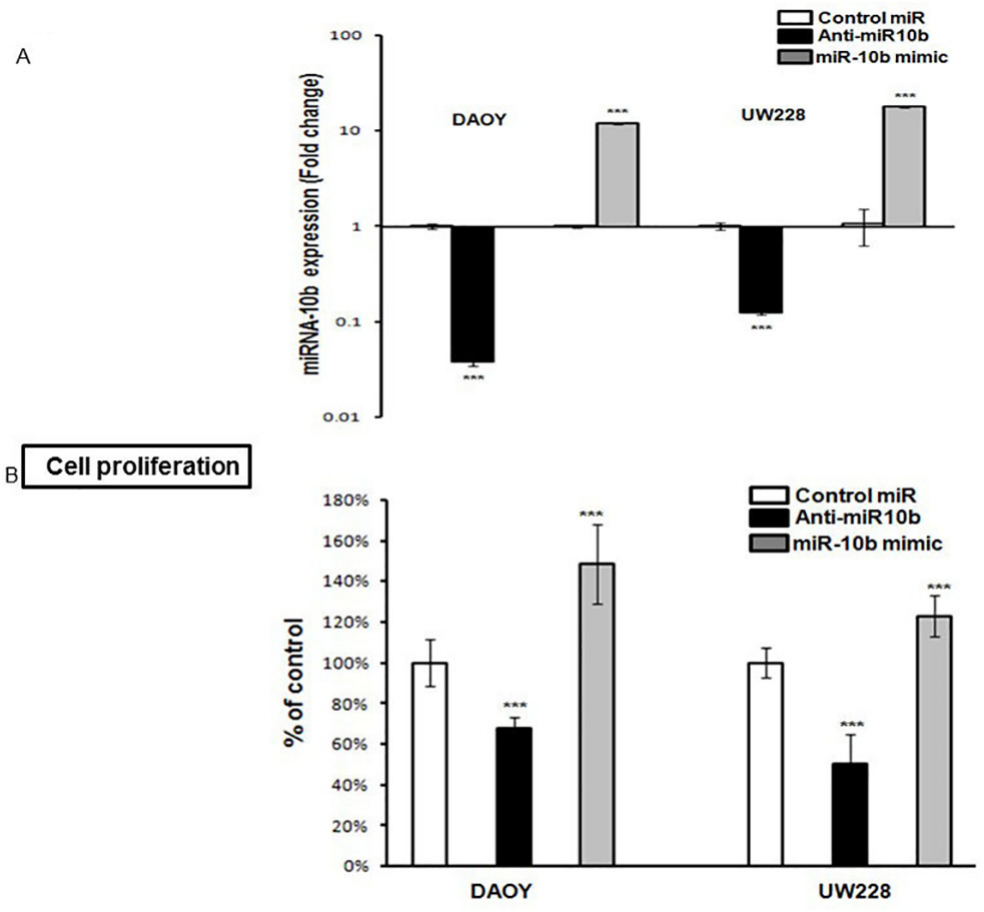
Inhibition of miRNA-10b inhibits cell proliferation and induces apoptosis. (A) Medulloblastoma cells were transfected with control miRNA, anti-miRNA-10b or miRNA-10b mimic. Total RNA was extracted after 48 hours of transfection and subjected to RT-PCR analysis for miRNA-10b expression. Error bars represent SD of the mean from at least 4 determinations. (B) Proliferation of transfected medulloblastoma cells with control miRNA, anti-miRNA-10b or miRNA-10b was analyzed by MTT proliferation assay measuring the intracellular NAD(P)H-oxidoreductase. (C) Percentage of apoptotic transfected cells was analyzed using a photometric cell death detection ELISA for identification of cytoplasmic histone-associated DNA fragments (n = 4). All results are represented as mean ± SD. (D) For the clonogenic growth assay, DAOY cells were transfected with miRNA-10b or inhibitor. After 24 hours, cells were seeded in 6-well plates (3000 cells/ well in triplicate). The plates were stained with the Hema 3 manual system after 12 to 14 days of incubation at 37°C. *Left*: representative photographs of the colonies; *right*: graphical representation of the colonies with over 50 cells ((** *P ≤ 0*.*05* and *** *P ≤ 0*.*001* according to student’s t-test).

To investigate the effects of miRNA-10b inhibition on medulloblastoma cells, DAOY and UW288 cells were transfected with control miRNA, anti-miRNA10b or miRNA-10b mimic. RT-PCR analysis showed that transfection of anti-miRNA10b significantly (P ≤ 0.001) inhibited the endogenous expression of miRNA-10b in both cell lines as compared to the control miRNA (Fig 2A). Transfection of miRNA10b significantly (P ≤ 0.001) upregulated miRNA10b expression in DAOY (12.55-fold compared to the control) and UW 228 (18.32-fold compared to the control) cells (Fig 2A), evidence that the transfection system is functioning well. Thus, manipulating the expression of miRNA-10b can be utilized to elucidate the functional effects of miRNA-10b on medulloblastoma cellular growth and survival.

The effects of miRNA-10b on proliferation and apoptosis induction in medulloblastoma were explored.). As miRNA-10b promotes cellular proliferation, transfection with miR-10b mimic was expected to promote proliferation and have little effect on apoptosis, while transfection with anti-miR-10b was expected to produce apoptosis and inhibit prolferation. These hypotheses were confirmed with the following investigations. Transfection of DAOY and UW228 MB cell lines with anti-miRNA-10b resulted in significant (P ≤ 0.001) inhibition of proliferation compared with cells transfected with control miRNA (Fig 2B). This was measured by MTT proliferation assay by quantifying the intracellular NAD(P)H-oxidoreductase. Transfection with miRNA-10b mimic significantly induced proliferation in both medulloblastoma cell lines (Fig 2B). The role of inhibition of miRNA-10b in the induction of medulloblastoma apoptosis was assessed. Medulloblastoma cells were transfected with control-miRNA, anti-miRNA10b or miRNA-10b mimic, and were analyzed by cell death detection ELISA. Significant induction of apoptosis by anti-miRNA-10b (*P* ≤ 0.001) (158.07% ± 5.38) andslightly decreased apoptosis in the presence of miRNA-10b mimic (97.20% ± 9.33) compared to control miRNA (100% ± 2.87) were observed in DAOY cells (n = 4 experiments; Fig 2C). A more significant (*P ≤* 0.001) pattern was observed in the UW228 cell line, with anti-miRNA10b (335.94% ± 17.68) and miRNA10b mimic (93.47% ± 5.70) compared to control miRNA (100% ± 6.53) (Fig 2C) The effect of the inhibition of miRNA10b on tumor cell growth was examined. DAOY and UW228 cells were transfected with control miRNA, anti-miRNA10b or miRNA-10b mimic for 48 hours. The transfected cells were plated for clonogenic growth. As shown in Fig 2D, miRNA-10b inhibition significantly inhibited clonogenic cell growth in both medulloblastoma cell lines. In the DAOY cell line, miRNA10b inhibition reduced colony formation by almost 70% as compared to control miRNA (58.66 ± 4.71 / 170 ± 5). In contrast, miRNA-10b mimic induced a20% increase in clonogenic growth compared to control (204.33± 6.02 / 170 ± 5) (Fig 2D). The same pattern was observed for the UW228 cell line for clonogenic growth (data not shown). Our data suggests that inhibition of miRNA-10b in medulloblastoma decreases proliferation via the induction of apoptosis, resulting in significant abrogation of clonogenic growth. In contrast, overexpression of miRNA-10b enhances proliferation and clonogenic growth by preventing apoptosis, implicating miRNA-10b in medulloblastoma proliferation and survival.

### Relationship between miRNA-10b and Bcl-2 expression

**Fig 3:**
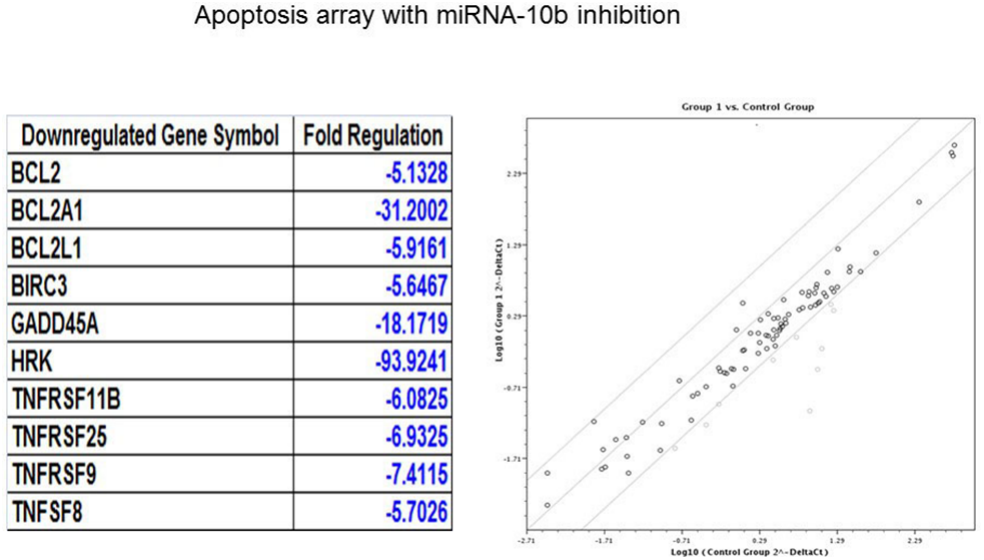
Anti-apoptotic BCL2 family members are downregulated by inhibition of miRNA-10b. (A) A human apoptosis array was used for the identification of apoptotic genes influenced by the inhibition of miRNA-10b. Total RNA was extracted from DAOY cells transfected with anti-miRNA-10b, and then subjected to RT-PCR for the analyses of apoptotic genes. The pale grey in the figure at right references the genes identified in the table at left. (B) Medulloblastoma cells were transfected with control miRNA, anti-miRNA-10b or miRNA-10b mimic. Lysates of transfected cells were subjected to Western blotting using (A) anti-BCL2 antibody (B) anti-MCL1 antibody. β-actin was used as a loading control.

We hypothesize that induction of apoptosis may be a major pathway by which miRNA-10b inhibition decreases the proliferation and survival of medulloblastoma cells. To investigate the modulation of apoptotic proteins by miRNA-10b, we performed a human apoptosis PCR array under the influence of miRNA-10b inhibitor transfection. Interestingly, we found in the gene array (Fig 3A) studies that inhibition of miRNA-10b significantly downregulated Bcl2 (-5.13 fold change) and anti-apoptotic Bcl2 family members such as BCL2A1 and BCL2L1. Transcriptional induction of Bcl2 is a major mechanism for apoptosis evasion in cancer cells. We found that inhibition of miRNA-10b resulted in significant inhibition of endogenous BCL2 and (anti-apoptotic) MCL-1 protein expression, and that overexpression of miRNA-10b increased expression of BCL2 and MCL-1 by western blot analysis (Fig 3B), suggesting that Bcl2 is under the control of miRNA-10b. There was no effect observed on the expression of pro-apoptotic BCL2 family members Bax, Bim and Bid (S1 Fig), which suggests that miRNA-10b is specifically targeting the expression of Bcl2 for the induction of proliferation in medulloblastoma. The 94-fold reduction in HRK expression is notable. This pro-apoptotic protein was not deemed a likely candidate for promoting proliferation in medulloblastoma, but this finding will be pursued in further studies. These data suggest that high levels of BCL2 may play a role in increasing the survival of medulloblastoma cells, and that miRNA-10b inhibits apoptosis and induces proliferation by targeting BCL2, an anti-apoptotic protein.

### Silencing of BCL2 also downregulates miRNA-10b expression

**Fig 4:**
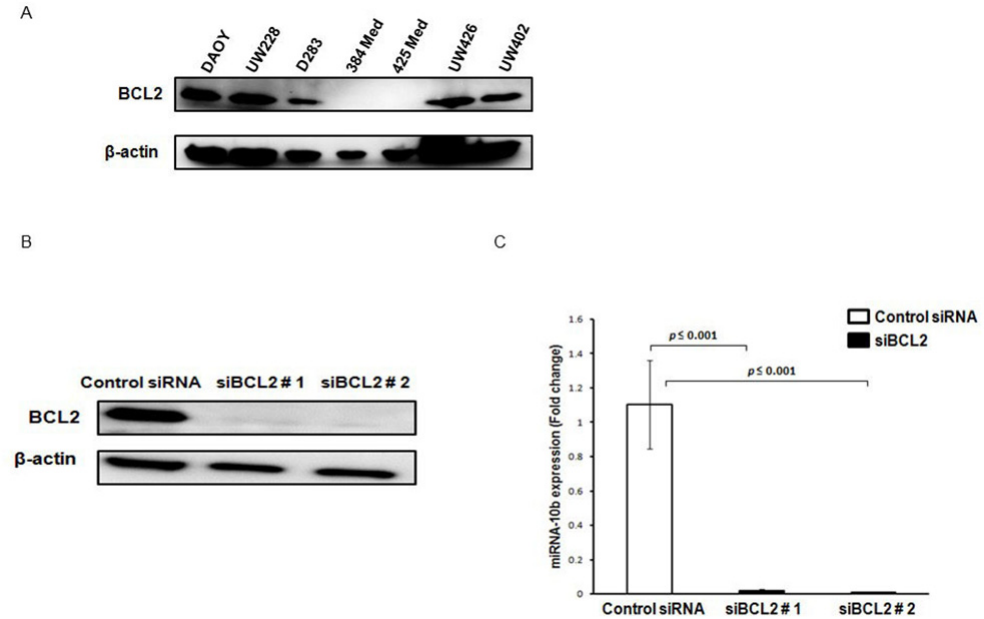
Knockdown of BCL2 by specific siRNA induces significant inhibition of miRNA-10b expression. DAOY, UW228, D283 384 Med, 425 Med, UW426 and UW402 medulloblastoma cells were collected and lysed, and cell lysates were fractionated by sodium dodecyl sulfate polyacrylamide gel electrophoresis (SDS-PAGE) and transferred onto PVDF membranes to determine BCL2 protein expression by Western blot analysis. The membrane was stripped and reprobed with an anti-β-actin antibody to confirm equal loading of protein in each lane. (B) DAOY cells were transfected with two different siRNAs (# 1 and #2) (10nM) specific for the BCL2 transcript, or with scrambled universal negative control RNA duplex (DS Scrambled Neg) (10nM). After 48 hours, cells were collected and BCL2 and β-actin expression was determined. (C) After 48 hours of BCL2 and negative control siRNA (10nM) treatment, total RNA was extracted, and mRNA levels of miRNA-10b were determined by RT-PCR analysis as described in Materials and Methods. Experiments were repeated at least three times. (** *P ≤ 0*.*05* and *** *P ≤ 0*.*001* according to student’s t-test)

It has been previously reported that downregulation of BCL2 by antisense oligonucleotides or siRNAs leads to apoptosis, and sensitizes cancer cells to chemotherapy and radiation therapy [20–24]. It was hypothesized that downregulation of BCL2 would downregulate miRNA-10b, and thus induce apoptosis and growth inhibition in medulloblastoma cells. BCL2 expression was assessed in the same group of 8 medulloblastoma cell lines which had been previously assessed for the expression of miRNA-10b (Fig 1B) and it was found that the cell lines with high levels of miRNA-10b expression also had high levels of Bcl2 expression. DAOY and UW228, the two cell lines which were used for all of the experiments in this study, showed very high expression of both miRNA -10b and BCL2 (Fig 4A). DAOY cells were used in this experiment. Treatment of DAOY cells with two different BCL2 siRNAs (10 nM) resulted in reduction of BCL2 protein expression of 75to 85% relative to control cells, as shown by Western blot performed at 48h of treatment (Fig 4B). The downregulation of BCL2 by BCL2 siRNA was demonstrated to inhibit the endogenous expression of miRNA-10b as detected by RT-PCR (Fig 4C). These findings suggest that apoptotic induction after miRNA-10b blockade is dependent to a significant degree on BCL2 downregulation.

### Down-regulation of miRNA-10b by potent BCL2 inhibitors

**Fig 5:**
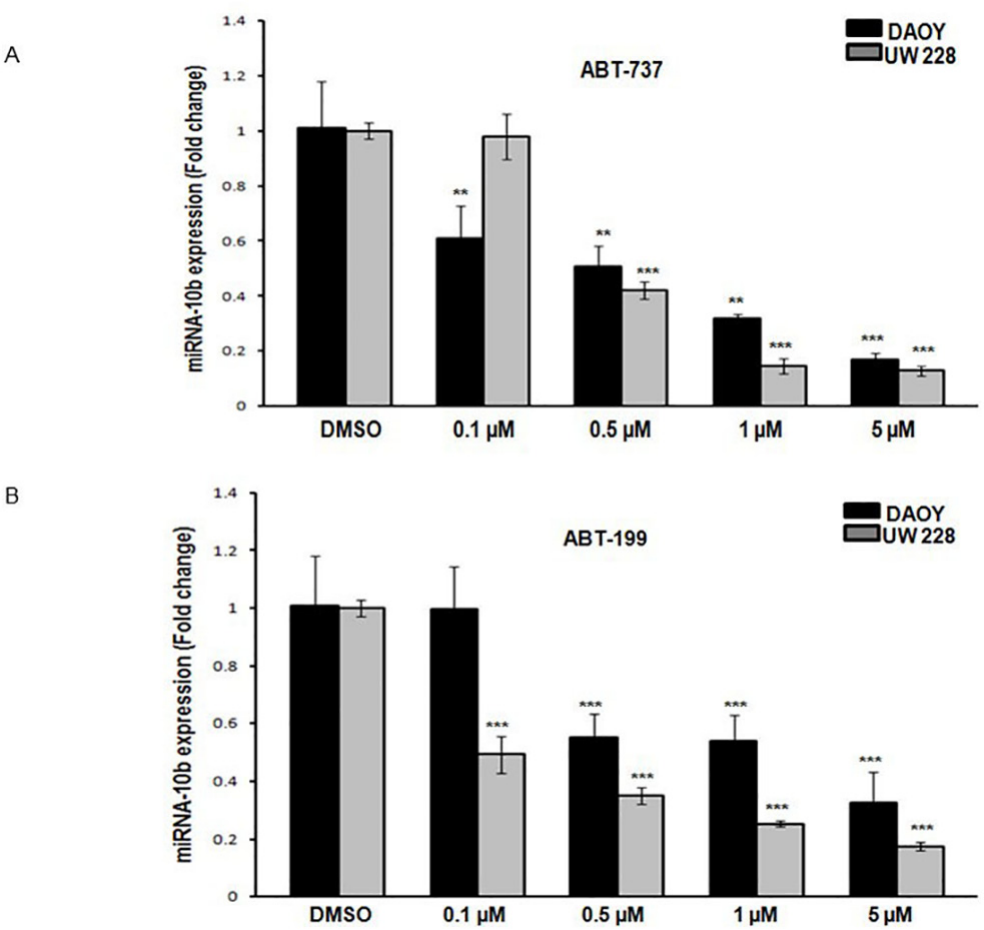
Down-regulation of miRNA-10b by BCL2 inhibitors is associated with the inhibition of proliferation by induction of apoptosis. Medulloblastoma cells were incubated with BCL2 inhibitors ABT-737 and ABT-199 for 48 hours. DMSO 0.1% was used as a control treatment. Total RNA was extracted from DAOY and UW228 cells, and subjected to RT-PCR for the analysis of miRNA-10b expression under the influence of BCL2 inhibitors (A) ABT-737 and (B) ABT-199. (C) ABT-737 and ABT-199 significantly inhibit the proliferation of medulloblastoma cells measured by MTT assay (D) ABT-737 and ABT-199 significantly induce the apoptosis of medulloblastoma cells measured by cell death detection ELISA. (** *P ≤ 0*.*05* and *** *P ≤ 0*.*001* according to student’s t-test)

Our preliminary studies indicated that miRNA-10b is an oncomir regulating the growth and survival of medulloblastoma cells by controlling the levels of BCL2. It was hypothesized that potent inhibitors of BCL2, known for their antiproliferative and apoptotic effects on medulloblastoma cells, inhibit the expression of miRNA-10b [25]. DAOY and UW228 cells were cultured with ABT-737 and ABT-199 as described in “Cell lines and reagents.” DMSO was used as a control. ABT-737, a BCL2 antagonist, has been shown to inhibit proliferation and induce apoptosis in medulloblastoma cells at 10 μM [25]. Concentrations ranging from 0.5 μM -10 μM for ABT-737 and ABT-199 were tested. Both ABT-737 and ABT-199 significantly (*P ≤* 0.001) inhibited endogenous miRNA-10b expression (Fig 5A and 5B) in a dose-dependent manner. ABT-737 and ABT-199 also significantly inhibited the proliferation of medulloblastoma cells as measured by MTT assay (Fig 5C), and induced apoptosis (Fig 5D), both in a dose-dependent manner.

The specificity of the inhibition of miRNA-10b expression by the BCL2 inhibitors was investigated. The BCL2 inhibitors ABT-737 and ABT-199 had no inhibitory effects on miRNA-17 and miRNA-20b expression (S2 Fig); these miRNAs were investigated because they were significantly upregulated in the cancer miRNA array (Fig 1A). These results suggest that miRNA-10b is specifically inhibited by the BCL2 inhibitors. To our knowledge this is the first study reporting the effects of ABT-199 on the growth and survival of medulloblastoma cells. This study suggests that BCL2 is an important mediator of miRNA-10b activity in medulloblastoma, and suggests a new therapeutic target.

### miRNA-10b expression correlates with survival and recurrence in patients with medulloblastomas

The correlation between the expression of miRNA-10b *in vivo* and the disease course in patients with medulloblastoma was investigated. miRNA-10b levels were measured in 13 primary medulloblastoma samples, and compared to a normal cerebellar control. As shown in Table 1, elevated levels of miRNA-10b expression (13–550 fold) compared to the control (*P ≤* 0.001) were detected in 4 primary medulloblastoma samples. The 2 patients with the highest levels of miRNA-10b expression (more than a 200-fold increase versus control) had multiple recurrences (an average of 4.5) and died within 8 years of diagnosis, compared with the 11 patients with lower levels of miRNA-10b expression who had a mean of 1.2 recurrences and 36% long-term survival (Table 1). The molecular subtype classification of the tumor samples was graciously provided by the laboratory of Michael D Taylor (Hospital for Sick Children, Toronto, Canada). The age, gender, MIB-1 proliferation index, pathologic subtype and molecular subgroups of the patients were not correlated with the levels of miRNA-10b expression. One patient with high expression of miRNA-10b had a tumor classified as being in the Wnt subgroup. Our laboratory studies were performed on the Daoy (SHH subgroup) and UW228 (group 3) cell lines, the lines with the highest levels of miRNA-10b expression. These facts are evidence that the role of miRNA-10b is more generalized and basic in the disease process, rather than having an effect in only a certain subgroup of tumors. Patients with high levels of miRNA-10b expression had poor survival and multiple tumor recurrences when compared to the patients with lower levels of miRNA-10b expression, correlating with our laboratory studies that indicate that miRNA-10b plays a role in medulloblastoma proliferation and inhibits apoptosis. Targeting of miRNA-10b with therapeutic intervention may lead to success in a large subset of patients, as miRNA-10b expression is not restricted to any known disease classification for medulloblastoma.

## Discussion

Medulloblastoma is the most common malignant brain tumor in children, and is associated with significant morbidity and mortality [26]. Current treatment methods are not tailored to the individual tumor, and convey significant long-term morbidity to the surviving patient [27,28]. Approximately one-third of patients remain refractory to therapy [29]. Resistance to therapy and relapse are hypothesized to be due to the deregulation of apoptotic factors such as the BCL2 family members [30]. These proteins are key regulators of apoptosis in the cerebellar granule cell precursors from which medulloblastoma are thought to arise [31,32]. miRNAs have been shown to target oncogenes to regulate cellular proliferation and survival [5,33,34], and have been implicated in the pathogenesis of medulloblastoma [35]. miRNA-10b has been shown to play an important role in the regulation of cell survival and proliferation [16], and to function as an oncomir in various types of malignancies [13,14,19]. This study demonstrates that miRNA -10b regulates the proliferation and survival of medulloblastoma cells by targeting BCL2. From an oncomir microarray, we demonstrated that miRNA-10b is one of the most highly upregulated miRNAs in medulloblastoma cell lines compared to the normal cerebellum. We observed that overexpression of miRNA-10b induces cellular proliferation and protects against apoptosis in medulloblastoma cell lines. Inhibition of miRNA-10b significantly induced apoptosis, resulting in the inhibition of proliferation of medulloblastoma cells. The Wnt and Sonic hedgehog pathways are known to have downstream effects on BCL2 transcription [36,37,38]. These regulatory effects may be mediated in some cases by miRNA-10b. These data suggest that therapies targeting miRNA-10b may be effective in the treatment of some or all of the molecular subgroups of medulloblastoma patients.

Our data indicates that the apoptosis induced by miRNA-10b blockade seems to be caused at least in part by changes in the expression of the anti-apoptotic protein BCL2. We found that endogenous levels of BCL2 are very well-correlated with miRNA-10b expression. DAOY and UW228, the cell lines with the highest levels of miRNA-10b expression, also showed the highest levels of BLC2 expression. In contrast, 384 Med and 425 Med, the cell lines with no expression of miRNA-10b, also showed no BCL2 expression. We found that overexpression of miRNA-10b significantly induced the expression of BCL2 in both the DAOY and UW228 cell lines, suggesting that miRNA-10b positively regulates BCL2 in medulloblastoma cells. In contrast, inhibition of miRNA-10b by anti-miRNA10b resulted in decreased BCL2 expression, and was accompanied by increased sensitivity of medulloblastoma cells to apoptosis. Our data provide strong evidence that miRNA-10b regulates BCL2 expression, and thus induces the proliferation of medulloblastoma cells. This mechanism could be responsible for resistance to chemotherapeutic drugs in some cases [39].

To further define the role BCL2 plays in apoptosis induced by miRNA-10b inhibition, we inhibited BCL2 expression in medulloblastoma cells, which resulted in significant downregulation of miRNA-10b expression. This indicates that miRNA-10b is regulated by BCL2, and miRNA-10b blockade seems to be caused at least in part by changes in the expression of the anti-apoptotic protein BCL2. This is further supported by the fact that two medulloblastoma cell lines with no endogenous expression of miRNA-10b also have no BCL2 expression, suggesting that high levels of miRNA-10b increase BCL2 levels, thereby increasing the proliferation and survival of medulloblastoma cells.

Our data showed that by regulating the anti-apoptotic protein BCL2, miRNA-10b produces oncogenic effects. Because BCL-2 inhibition downregulated miRNA-10b expression, we investigated whether BCL2 inhibitors could be used to inhibit the effects of miRNA-10b. ABT-737 is a BCL2 homology 3 (BH3)-mimetic that induces apoptosis by inhibiting pro-survival BCL2 proteins [40]. High levels of BCL2 were shown to correlate with ABT-737 sensitivity in CLL [41,42]. ABT-737 is under early-phase clinical evaluation as single agent therapy for CLL at present, but preclinical studies highlight its potential for use in combined modality therapies [43]. The main obstacle to its implementation appears to be a side effect of thrombocytopenia. Levesley et al showed that ABT-737 induces apoptosis in medulloblastoma cells [25]. We found that ABT-737 inhibits miRNA-10b expression in a dose-dependent fashion, and that this is accompanied by the inhibition of cell proliferation and increased induction of apoptosis. Recently Souers et al[44] showed that ABT-199 is a first-in-class orally bioavailable BCL2 selective inhibitor that shows potent cell killing *in vitro* and high antitumor efficacy *in vivo*. In our study, ABT-199 inhibited medulloblastoma cellular proliferation by induction of apoptosis in a dose- dependent fashion, and also inhibited miRNA-10b expression.

To our knowledge, we are the first to report that miRNA-10b is involved in the regulation of proliferation and survival in medulloblastoma. Our findings on the role of miRNA-10b in medulloblastoma are in agreement with previous reports describing its role in the proliferation and maintenance of glioma cells by regulating various apoptotic proteins [16]. Gabriely et al suggested that miRNA-10b has effects upstream of the cell cycle and antiapoptotic genes, controlling major pathway divergence points between proliferation and cell death in glioma [16]. Of note, miR-34a expression was downregulated on our initial oncomir microarray (Fig 1), inversely correlated with miR-10b as it has been reported to be with Bcl-2 expression in gliomas. [45] The effect of miR-10b on Bcl-2 may occur through a loss of miR-34a in medulloblastoma. We are the first group to report upon the inhibition of miRNA expression by the BCL2 inhibitors ABT-737 and ABT-199. The ongoing clinical evaluation of BCL-2 inhibitors and this new knowledge of the effects of these drugs on miRNA-10b expression may broaden their application as a new chemotherapeutic class, an anti-miRNA drug.

Kaplan-Meier survival analysis available through the TCGA Cancer Molecular Analysis Portal [46] suggested that miRNA-10b expression may correlate with patient survival [47]. Our patient data showed that patients with high miRNA-10b levels have much shorter survival and more tumor recurrences compared with patients with low miRNA-10b expression. The fact that the patient data correlated with miRNA-10b expression but not with their molecular subgroup classification argues that the use of miRNA-10b as a biomarker is more broadly applicable than to simply identify a single molecular subgroup. Our data strongly support the oncogenic role of miRNA-10b in medulloblastoma cellular proliferation.

In summary, we describe the functional role of miRNA-10b in medulloblastoma. We demonstrate that high levels of miRNA-10b increase medulloblastoma proliferation by directly or indirectly regulating BCL2 levels, thereby controlling apoptosis. The mechanistic steps between miRNA-10b and BCL2 in medulloblastoma have yet to be elucidated. Our data suggest that regulation of BCL2 by miRNA-10b is associated with proliferation and survival of medulloblastoma cells.

## Supporting Information

S1 Fig Pro-apoptotic BCL-2 family members are not affected by miRNA-10b expression. Medulloblastoma cells were transfected with control miRNA, anti-miRNA-10b, or miRNA-10b mimic. Lysates of transfected cells were subjected to Western blotting using anti-Bim, anti-Bcl-xl, anti-Bid, and anti-Bax antibodies. β-actin was used as a loading control. Thus, the effect of miRNA-10b expression on BCL-2 is specific to certain BCL-2 family members.
(TIFF)

S2 Fig The effect of BCL2 inhibition is specific to miRNA-10b. Medulloblastoma cells (DAOY) were incubated with the BCL2 inhibitors ABT-737 and ABT-199 for 48 hours. DMSO 0.1% was used as control treatment. Total RNA was extracted from DAOY cells and subjected to RT-PCR for the analysis of (A) miRNA-17 and (B) miRNA-20b expression under the influence of BCL2 inhibitors ABT-737 and ABT-199. Error bars represent SD from the mean from at least 4 repeat experiments. This demonstrates that the effect of BCL2 inhibition on miRNA-10b is specific to miRNA-10b.
(TIFF)

## References

[1] Taylor MD, Northcott PA, Korshunov A, Remke M, Cho YJ, Clifford SC. et al. (2011) Molecular subgroups of medulloblastoma: the current consensus. Acta Neuropathol 123: 465–472. 10.1007/s00401-011-0922-z 22134537 PMC3306779

[2] Keles GE, Berger MS, Srinivasan J, Kolstoe DD, Bobola MS, Silber JR. (1995) Establishment and characterization of four human medulloblastoma-derived cell lines. Oncol Res 7 (10–11): 493–503. 8866661

[3] Bigner SH, Friedman HS, Vogelstein B, Oakes WJ, Bigner DD. (1990) Amplification of the c-myc gene in human medulloblastoma cell lines and xenografts. Cancer Res 50: 2347–2350. 2180567

[4] Cho WC. (2010) MicroRNAs: potential biomarkers for cancer diagnosis, prognosis and targets for therapy. Int J Biochem Cell Biol 42(8): 1273–81. 10.1016/j.biocel.2009.12.014 20026422

[5] Cho WC. (2010) MicroRNAs in cancer—from research to therapy. Biochim Biophys Acta 1805(2): 209–17. 10.1016/j.bbcan.2009.11.003 19931352

[6] Ma L and Weinberg RA.(2008) Micromanagers of malignancy: role of microRNAs in regulating metastasis. Trends Genet 24(9): 448–56. 10.1016/j.tig.2008.06.004 18674843

[7] Nicoloso MS, Spizzo R, Shimizu M, Rossi S, Calin GA. (2009) MicroRNAs—the micro steering wheel of tumour metastases. Nat Rev Cancer 9(4): 293–302. 10.1038/nrc2619 19262572

[8] Hammond SM. (2006) MicroRNAs as oncogenes. Curr Opin Genet Dev 16(1):4–9. 16361094 10.1016/j.gde.2005.12.005

[9] Hwang HW, Mendell JT. (2006) MicroRNAs in cell proliferation, cell death, and tumorigenesis. Br J Cancer 94(6): 776–80. 16495913 10.1038/sj.bjc.6603023 PMC2361377

[10] Venkataraman S, Alimova I, Fan R, Harris P, Foreman N, Vibhakar R. (2010) MicroRNA 128a increases intracellular ROS level by targeting Bmi-1 and inhibits medulloblastoma cancer cell growth by promoting senescence. PLoS One 5(6): e10748.11. 20574517 10.1371/journal.pone.0010748 PMC2888574

[11] Grunder E, D'Ambrosio R, Fiaschetti G, Abela L, Arcaro A, Zuzak T, et al (2011) MicroRNA-21 suppression impedes medulloblastoma cell migration. Eur J Cancer 47(16): 2479–90. 10.1016/j.ejca.2011.06.041 21775132

[12] Ciafre SA, Galardi S, Mangiola A, Ferracin M, Liu CG, Sabatino G, et al (2005) Extensive modulation of a set of microRNAs in primary glioblastoma. Biochem Biophys Res Commun 334(4):1351–8. 16039986 10.1016/j.bbrc.2005.07.030

[13] Sasayama T, Nishihara M, Kondoh T, Hosoda K, Kohmura E. (2009) MicroRNA-10b is overexpressed in malignant glioma and associated with tumor invasive factors, uPAR and RhoC. Int J Cancer 125(6):1407–13. 10.1002/ijc.24522 19536818

[14] Ma L, Teruya-Feldstein J, Weinberg RA. (2007) Tumour invasion and metastasis initiated by microRNA-10b in breast cancer. Nature 449(7163): 682–8. 17898713 10.1038/nature06174

[15] Garzon R, Garofalo M, Martelli MP, Briesewitz R, Wang L, Fernandez-Cymering C, et al (2008) Distinctive microRNA signature of acute myeloid leukemia bearing cytoplasmic mutated nucleophosmin. Proc Natl Acad Sci U S A. 105(10): 3945–50. 10.1073/pnas.0800135105 18308931 PMC2268779

[16] Gabriely G, Yi M, Narayan RS, Niers JM, Wurdinger T, Imitola J, et al (2011) Human glioma growth is controlled by microRNA-10b. Cancer Res 71(10): 3563–72. 10.1158/0008-5472.CAN-10-3568 21471404 PMC3096675

[17] Tian Y, Luo A, Cai Y, Su Q, Ding F, Chen H, et al (2010) MicroRNA-10b promotes migration and invasion through KLF4 in human esophageal cancer cell lines. J Biol Chem 285(11): 7986–94. 10.1074/jbc.M109.062877 20075075 PMC2832949

[18] Chai G, Liu N, Ma J, Li H, Oblinger JL, Prahalad AK, et al (2010) MicroRNA-10b regulates tumorigenesis in neurofibromatosis type 1. Cancer Sci 101(9):1997–2004. 10.1111/j.1349-7006.2010.01616.x 20550523 PMC11159772

[19] Garzon R, Croce CM. (2008) MicroRNAs in normal and malignant hematopoiesis. Curr Opin Hematol 15(4): 352–8. 10.1097/MOH.0b013e328303e15d 18536574

[20] Kitada S, Takayama S, De Riel K, Tanaka S, Reed JC. (1994) Reversal of chemoresistance of lymphoma cells by antisense-mediated reduction of bcl-2 gene expression. Antisense Res Dev 4(2): 71–9. 7950302 10.1089/ard.1994.4.71

[21] Poeck H, Besch R, Maihoefer C, Renn M, Tormo D, Morskaya SS, et al (2008) 5'-Triphosphate-siRNA: turning gene silencing and Rig-I activation against melanoma. Nat Med 14(11): 1256–63 10.1038/nm.1887 18978796

[22] Konopleva M, Tari AM, Estrov Z, Harris D, Xie Z, Zhao S, et al (2000) Liposomal Bcl-2 antisense oligonucleotides enhance proliferation, sensitize acute myeloid leukemia to cytosine-arabinoside, and induce apoptosis independent of other antiapoptotic proteins. Blood 95(12): 3929–38. 10845930

[23] Yde CW, Issinger OG. (2006) Enhancing cisplatin sensitivity in MCF-7 human breast cancer cells by down-regulation of Bcl-2 and cyclin D1. Int J Oncol 29(6): 1397–404. 17088977

[24] Lima R T, Martins L, M., Guimaraes J E, Sambade C, Vasconcelos MH(2004) Specific downregulation of bcl-2 and xIAP by RNAi enhances the effects of chemotherapeutic agents in MCF-7 human breast cancer cells. Cancer Gene Ther 11 (5): 309–316. 15031723 10.1038/sj.cgt.7700706

[25] Levesley J, Lusher ME, Lindsey JC, Clifford SC, Grundy R, Coyle B. (2011) RASSF1A and the BH3-only mimetic ABT-737 promote apoptosis in pediatric medulloblastoma cell lines. Neuro Oncol 13 (12); 1265–1276). 21880625 10.1093/neuonc/nor129 PMC3223089

[26] Gilbertson RJ. (2004) Medulloblastoma: signalling a change in treatment. Lancet Oncol 5 (4): 209–218. 15050952 10.1016/S1470-2045(04)01424-X

[27] Stavrou T, Bromley CM, Nicholson HS, Byrne J, Packer RJ, Goldstein AM, et al. (2001) Prognostic factors and secondary malignancies in childhood medulloblastoma. J Pediatr Hematol Oncol 23(7):431–6. 11878577 10.1097/00043426-200110000-00008

[28] Packer RJ, Cogen P, Vezina G, Rorke LB. (1999) Medulloblastoma: clinical and biologic aspects. Neuro Oncol. 1(3):232–50. 11550316 10.1215/15228517-1-3-232 PMC1920747

[29] Gilbertson RJ, Ellison DW (2008) The origins of medulloblastoma subtypes. Annu Rev Pathol 3:341–65. 18039127 10.1146/annurev.pathmechdis.3.121806.151518

[30] Lowe SW, Lin AW. (2000) Apoptosis in cancer. Carcinogenesis 21(3):485–95. 10688869 10.1093/carcin/21.3.485

[31] Lossi L, Zagzag D, Greco MA, Merighi A. (1998) Apoptosis of undifferentiated progenitors and granule cell precursors in the postnatal human cerebellar cortex correlates with expression of BCL-2, ICE, and CPP32 proteins. J Comp Neurol 399(3): 359–72. 9733083

[32] Tanabe H, Eguchi Y, Kamada S, Martinou JC, Tsujimoto Y. (1997) Susceptibility of cerebellar granule neurons derived from Bcl-2-deficient and transgenic mice to cell death. Eur J Neurosci 9(4): 848–56. 9153592 10.1111/j.1460-9568.1997.tb01434.x

[33] Esquela-Kerscher A, Slack FJ. (2006) Oncomirs—microRNAs with a role in cancer. Nat Rev Cancer 6(4): 259–69. 16557279 10.1038/nrc1840

[34] Calin G A, Croce CM. (2006) MicroRNA signatures in human cancers. Nat Rev Cancer 6(11): 857–66 17060945 10.1038/nrc1997

[35] Zhi F, Wang S, Wang R, Xia X, Yang Y. (2013) From small to big:microRNAs as new players in medulloblastomas. Tumour Biol 34(1): 9–15. 10.1007/s13277-012-0579-9 23179395

[36] Ming M, Wang S, Wu W, Senyuk V, Le Beau MM, Nucifora G, et al (2012) Activation of Wnt/beta-catenin protein signaling induces mitochondria-mediated apoptosis in hematopoietic progenitor cells. J Biol Chem 287(27): 22683–90. 10.1074/jbc.M112.342089 22589536 PMC3391123

[37] Pecina-Slaus N. (2010) Wnt signal transduction pathway and apoptosis: a review. Cancer Cell Int 10–22. 10.1186/1475-2867-10-22 20591184 PMC2908610

[38] Katoh Y Katoh M.(2009) Hedgehog target genes: mechanisms of carcinogenesis induced by aberrant hedgehog signaling activation. Curr Mol Med 9(7): 873–86. 19860666 10.2174/156652409789105570

[39] Blagoskionny MV. (2001) Paradox of Bcl-2 (and p53): why may apoptosis-regulating proteins be irrelevant to cell death? Bioessays 23(10): 947–53. 11598961 10.1002/bies.1135

[40] Rooswinkel RW, van de Kooij B, Verheij M, Borst J. (2012) Bcl-2 is a better ABT-737 target than Bcl-xL or Bcl-w and only Noxa overcomes resistance mediated by Mcl-1, Bfl-1, or Bcl-B. Cell Death Dis 3:e366 10.1038/cddis.2012.109 22875003 PMC3434657

[41] Del Gaizo Moore V, Schlis KD, Sallan SE, Armstrong SA, Letai A. (2008) BCL-2 dependence and ABT-737 sensitivity in acute lymphoblastic leukemia. Blood 111(4): 2300–9. 18056841 10.1182/blood-2007-06-098012 PMC2234061

[42] Al-Harbi S, Hill BT, Mazumder S, Singh K, Devecchio J, Choudhary G, et al (2011) An antiapoptotic BCL-2 family expression index predicts the response of chronic lymphocytic leukemia to ABT-737. Blood 118(13): 3579–90. 10.1182/blood-2011-03-340364 21772052 PMC3186334

[43] Cragg MS, Harris C, Strasser A, Scott CL. (2009) Unleashing the power of inhibitors of oncogenic kinases through BH3 mimetics. Nat Rev Cancer 9(5):321–6. 10.1038/nrc2615 19343035

[44] Souers AJ, Leverson JD, Boghaert ER, Ackler SL, Catron ND, Chen J, et al (2013) ABT-199, a potent and selective BCL-2 inhibitor, achieves antitumor activity while sparing platelets. Nat Med 19(2):202–8. 10.1038/nm.3048 23291630

[45] Gao H, Zhao H, Xiang W. (2013) Expression level of human miR-34a correlates with glioma grade and prognosis. J Neurooncol 113 (2): 221–8. 10.1007/s11060-013-1119-1 23529798

[46] TCGA Cancer Molecular Analysis Portal [cited 2011 Jan 24]. Available from: https://cma.nci.nih.gov/cma-tcga/.

[47] TCGA Cancer Molecular Analysis for miR-10b [cited 2011 Jan Available from https://cma.nci.nih.gov/cma-tcga/geneView/kmPlot?control_taskId¼1295909131920&taskId¼1295909131320&geArrayPlatform¼TCGA_miRNA_level2_Jan30_09.Rda&sampleGroups¼All_Patients&platformName¼MIRNA&reporter¼Median_of_All_Reporters&geneSymbol¼hsa-mir-10b&plotType¼GE_KM_PLOT&plot¼GE_KM_PLOT&method¼Go.

