# Supplementary figures and images for: microRNA-10b Is Overexpressed and Critical for Cell Survival and Proliferation in Medulloblastoma
Source: PLoS One. 2015 Sep 22;10(9):e0137845. doi: 10.1371/journal.pone.0137845 (PMC4579065; doi:10.1371/journal.pone.0137845)

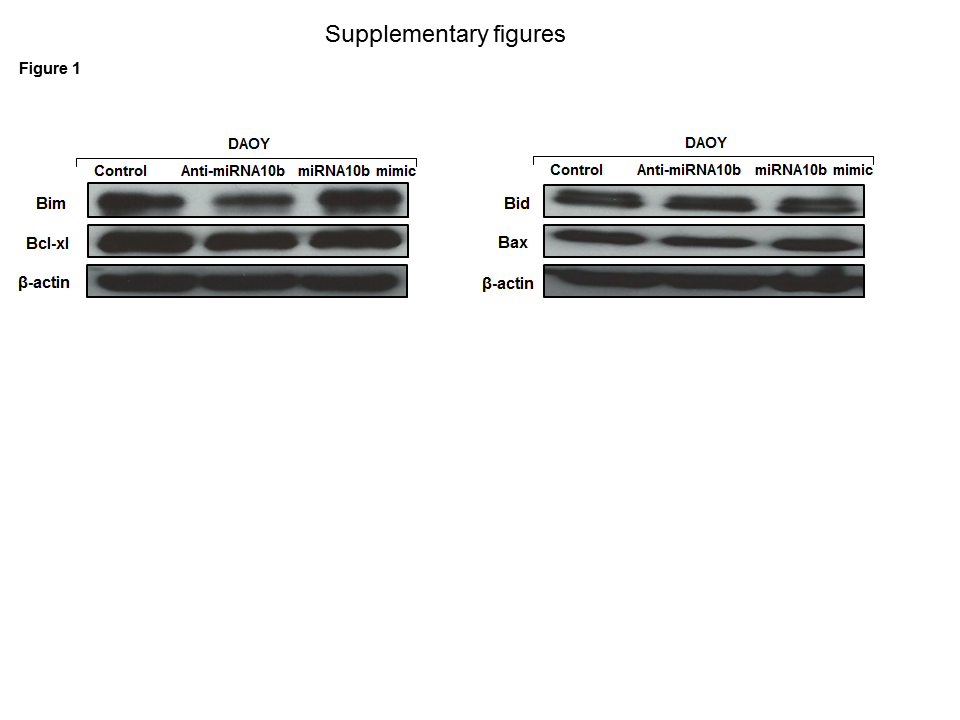

Supplement: S1 Fig — Medulloblastoma cells were transfected with control miRNA, anti-miRNA-10b, or miRNA-10b mimic. Lysates of transfected cells were subjected to Western blotting using anti-Bim, anti-Bcl-xl, anti-Bid, and anti-Bax antibodies. β-actin was used as a loading control. Thus, the effect of miRNA-10b expression on BCL-2 is specific to certain BCL-2 family members. (TIFF) [file pone.0137845.s001.tiff]

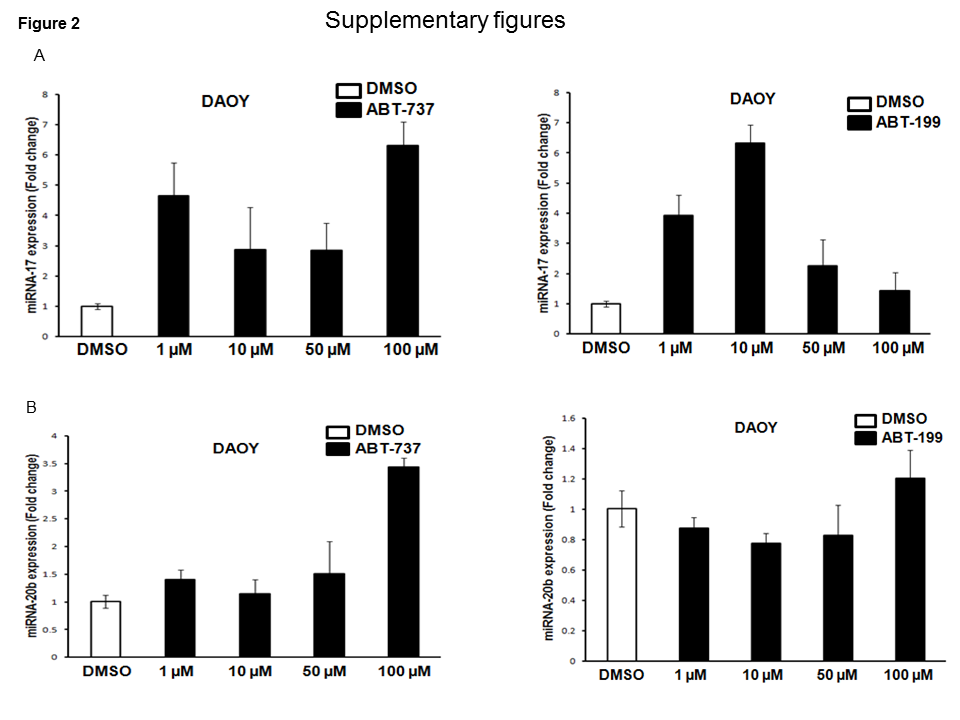

Supplement: S2 Fig — Medulloblastoma cells (DAOY) were incubated with the BCL2 inhibitors ABT-737 and ABT-199 for 48 hours. DMSO 0.1% was used as control treatment. Total RNA was extracted from DAOY cells and subjected to RT-PCR for the analysis of (A) miRNA-17 and (B) miRNA-20b expression under the influence of BCL2 inhibitors ABT-737 and ABT-199. Error bars represent SD from the mean from at least 4 repeat experiments. This demonstrates that the effect of BCL2 inhibition on miRNA-10b is specific to miRNA-10b. (TIFF) [file pone.0137845.s002.tiff]
